# Researching trends in pemphigoid diseases: A bibliometric study of the top 100 most cited publications

**DOI:** 10.3389/fmed.2022.1088083

**Published:** 2023-01-09

**Authors:** Shih-Cheng Huang, Tsu-Man Chiu, Chien-Ying Lee, Hui-Chin Chang, Wen-Jun Wu, Shuo-Yan Gau

**Affiliations:** ^1^Institute of Medicine, Chung Shan Medical University, Taichung, Taiwan; ^2^School of Medicine, Chung Shan Medical University, Taichung, Taiwan; ^3^Department of Dermatology, Chung Shan Medical University Hospital, Taichung, Taiwan; ^4^Department of Pharmacology, Chung Shan Medical University, Taichung, Taiwan; ^5^Department of Pharmacy, Chung Shan Medical University Hospital, Taichung, Taiwan; ^6^Library, Chung Shan Medical University Hospital, Taichung, Taiwan; ^7^Evidence-Based Medicine Center, Chung Shan Medical University Hospital, Taichung, Taiwan; ^8^Department of Microbiology and Immunology, School of Medicine, Chung Shan Medical University, Taichung, Taiwan; ^9^Department of Medical Research, Chung Shan Medical University Hospital, Taichung, Taiwan

**Keywords:** bibliometric analysis, pemphigoid diseases, dermatology, immunology, autoimmune

## Abstract

**Background:**

In the field of autoimmune and inflammatory disorders, different approaches were applied to provide information regarding disease activity, comorbidities, epidemiological reports and risk factors. However, no previous studies had thoroughly analyzed the research trend in the field, and the bibliometric analysis focusing on pemphigoid diseases was available. The objective of the current study was to evaluate the current research trend in the field.

**Methods:**

A search has been conducted for the Web of Science database based on various subcategories of pemphigoid diseases. Detailed information including articles’ publication types, Author information, citation, and publication information was attained for further analysis.

**Results:**

Within the 6,995 studies, the top 100 most-cited articles were extracted for analysis. Among the top 100 studies, 70% of the studies focused on bullous pemphigoid. More than 60% of the top 100 studies were studies with original data. Furthermore, 30% of the studies were guidelines and narrative reviews. For the issues primarily focused on, most of the high-impact studies described the molecular mechanism of pemphigoid diseases (26%), managements (19%), risk factors of pemphigoid diseases (17%). Additionally, some other studies provided general review or discussed about the issue of epidemiology, diagnosis/definition, comorbidities and clinical characteristics of pemphigoid diseases.

**Conclusion:**

This comprehensive bibliographic study of pemphigoid diseases provided an overview of current research focuses in the field. Topics such as disease management, molecular mechanism of pathogenesis, and drug-inducing pemphigoid diseases were highly mentioned in the most-cited studies. For researchers and clinicians, the researching trend and study focus in the top-100 cited studies could serve as a potential reference for future investigation and patient management.

## Introduction

Patients with pemphigoid diseases present with tense blisters and erosions. With autoimmune effects in cutaneous areas, the appearance of the patients would be affected and could significantly influence patient quality of life and psychological status (1). According to previous research, eight diseases were identified as subcategories of pemphigoid diseases, including bullous pemphigoid, mucous membrane pemphigoid, pemphigoid gestationis, linear IgA disease, epidermolysis bullosa acquisita, anti-p200 pemphigoid, and lichen planus pemphigoides (2). Given that the influence of pemphigoid diseases was gradually increasing, the burden caused by pemphigoid diseases could be underestimated (3). Under such circumstances, attention should be raised.

In the field of autoimmune and inflammatory disorders, researchers used different approaches to provide information on disease activity, comorbidities, epidemiological reports, and risk factors (4–6). Within the past 20 years, the definition criteria, diagnostic techniques, and management of pemphigoid diseases were constantly improving. In previous studies, study types including guidelines, reviews, original studies, and case studies were applied. However, to our knowledge, to date no previous studies had thoroughly analyzed the research trend in the field, and the bibliometric analyses focused on pemphigoid diseases were not available. Therefore, we conducted a bibliometric study analyzing the top 100 most cited studies regarding pemphigoid diseases to evaluate the current research trend in the field.

## Materials and methods

### Searching strategy

A search of the Web of Science database was conducted on 13 October 2022. The searching syntaxes were based on “Bullous pemphigoid” or “Mucous membrane pemphigoid” or “Cicatricial Pemphigoid” or “Pemphigoid gestationis” or “Linear IgA” or “Epidermolysis Bullosa Acquisita” or “anti-p200 pemphigoid” or “lichen planus pemphigoides or “pemphigoid”. The strategy of categorizing pemphigoid diseases in this study was also applied in previous studies (2). Regarding the time range of the data, no limitations were set. Detailed information including articles’ publication types (journal article or conference article), author information (full names, addresses, and affiliations), citation information (times cited in Web of Science core collection and all databases), publication information (publication year, date, volume, issue, starting page and ending page, DOI, PubMed ID, research areas, and open access designations) were attained from the Web of Science database on the same day of the research performed (13 October 2022). All analyzes were performed based on the basis of the data obtained to prevent biases caused by the update of the citation information. All included articles were sorted by citation number in the order of highest to the lowest. Only studies focusing primarily on pemphigoid diseases will be included in the study and extracted. Studies that met the following exclusion criteria were excluded from extraction and further analyzes: (1) studies not related to any pemphigoid disease; (2) studies mentioning pemphigoid diseases but focus primarily on other topics; and (3) animal studies. After critical appraisal of articles, eligible publications were ranked in the sequence of cited amounts. When multiple articles were cited the same times, articles with a recent year of publication will obtain a higher rank than those articles with relatively previous publication year.

### Data extraction and statistical analysis

Within the eligible studies, information about the 100 most cited studies about pemphigoid diseases was extracted. Basic information (year of publication, authors’ name, citation times of the article) and detailed information on the content (nation or region the study originated, study design, subcategories of pemphigoid disease each study focuses on, main finding of each study and impact factor 2021 of the journal publishing each article) were extracted for further analysis. Statistical analysis and figure examination were performed using Microsoft Excel 2019.

## Results

### Extracted studies

Within the 6,995 studies retrieved from the Web of Science, the top 100 most-cited articles were extracted for analysis. Detailed extracted information of the top 100 articles was available in Supplementary Table 2 (2, 7–105).

### Publication time trend and open access options

**FIGURE 1:**
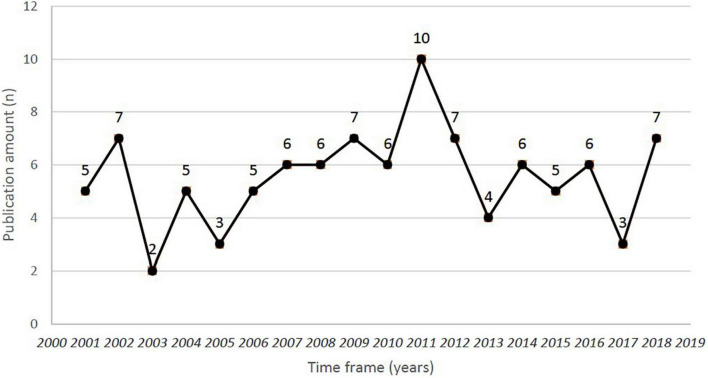
Time frame and publication amount for the top 100 most-cited studies of pemphigoid diseases.

**FIGURE 2:**
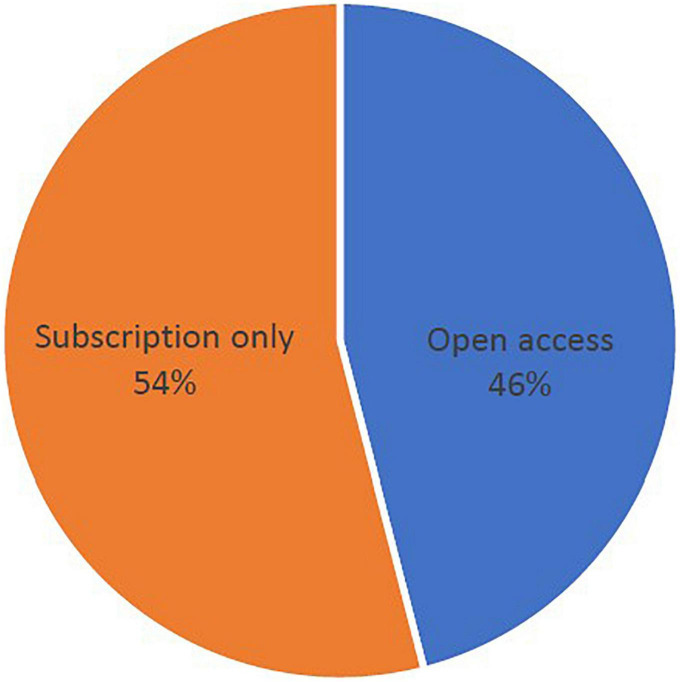
Open access status of the top 100 most-cited studies of pemphigoid diseases.

Within the top 100 publications of pemphigoid diseases that are the newest studies were published in 2018, while the most previous studies were published in 2001. The highest number of most cited publications was found in 2011, with the amount of 10 publications (Figure 1). However, the lowest number of articles in the top 100 publications was found in 2003, with the amount of 2 publications. The newest study of the top 100 publications was published in December 2018 in the *Journal of the American Academy of Dermatology*, entitled “*Bullous disorders associated with anti-PD-1 and anti-PD-L1 therapy: A retrospective analysis evaluating clinical and histopathologic features, frequency, and impact on cancer therapy*,” which was a single-center retrospective study evaluating the development of pemphigoid diseases (including bullous pemphigoid and linear IgA bullous) after cancer therapy (86). The oldest study was entitled “*Anti-epiligrin cicatricial pemphigoid and relative risk for cancer*” which was published in *The Lancet* in research letter form in June 2001. This retrospective American cohort study reported a 6.8-fold cancer risk in patients with cicatricial pemphigoid patients (32). Within the top-100 most cited studies, 54% of them were published based on traditional subscription model, whereas 46% of them published in open access (Figure 2).

### Citation amount

The number of the top 100 most cited studies of pemphigoid diseases ranged from 73 to 581 cited times. The accumulated number of cited studies of the top 100 was 12,149 times. On average, these articles were cited 121.49 times per article. Among the top 100 studies, a review article published in *The Lancet* in January 2013, entitled “*Pemphigoid diseases*” and conducted by Schmidt et al. was most cited (581 times).

### Journal distribution

In summary, 36 different journals have contributed to the top 100 most cited studies of pemphigoid diseases (Table 1). The *Journal of Investigative Dermatology* contributed 16 studies, which was the highest amount compared to other journals. The journals contributing more than five studies included the *British Journal of Dermatology* (*n* = 11), the *Journal of the American Academy of Dermatology* (*n* = 11), the *Archives of Dermatology* (*n* = 9), the *Clinics in Dermatology* (*n* = 5), and the *Journal of Dermatological Science* (*n* = 5).

**TABLE 1:** Journal information for the top 100 most-cited publications of pemphigoid diseases.

Journal names	Amount of top 100 publications	IF 2021^1^
*Journal of Investigative Dermatology*	16	7.59
*British Journal of Dermatology*	11	11.11
*Journal of the American Academy of Dermatology*	11	15.49
*Archives of Dermatology* ^2^	9	NA
*Clinics in Dermatology*	5	2.80
*Journal of Dermatological Science*	5	5.41
*JAMA Dermatology*	3	11.82
*Journal Der Deutschen Dermatologischen Gesellschaft*	3	5.23
*Lancet*	3	202.73
*Ophthalmology*	3	14.28
*Autoimmunity Reviews*	2	17.39
*Clinical Reviews in Allergy & Immunology*	2	10.82
*Experimental Dermatology*	2	4.51
*Journal of the European Academy of Dermatology and Venereology*	2	9.23
*American Journal of Clinical Dermatology*	1	6.23
*Annual Review of Pathology: Mechanisms of Disease*	1	32.35
*Archives of Dermatological Research*	1	3.03
*British Medical Journal*	1	93.33
*Cancer Immunology Research*	1	12.02
*Clinical and Experimental Dermatology*	1	4.48
*Clinical Immunology*	1	10.19
*Cochrane Database of Systematic Reviews*	1	12.01
*Dermatologic Clinics*	1	3.65
*Dermatology*	1	5.20
*European Journal of Dermatology*	1	2.81
*Frontiers in Medicine*	1	5.06
*International Journal of Dermatology*	1	3.20
*Journal of Allergy and Clinical Immunology*	1	14.29
*Journal of Cellular and Molecular Medicine*	1	5.30
*Journal of Clinical Investigation*	1	19.46
*Journal of Cutaneous Pathology*	1	1.46
*Journal of Immunology*	1	5.43
*Medicine*	1	1.82
*Melanoma Research*	1	3.20
*Proceedings of the National Academy of Sciences of the United States of America*	1	12.78
*The American Journal of Pathology*	1	5.77
*The New England Journal of Medicine*	1	176.08

^1^According to the journal citation report 2021 published by Clarivate.

^2^“Archives of Dermatology” has changed its name into “JAMA Dermatology” at January, 2013.

### Country of origin

**FIGURE 3:**
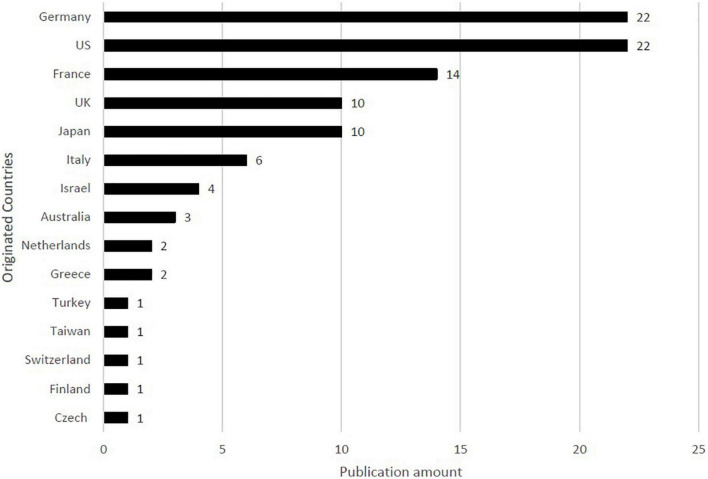
Countries and regions the top 100 most-cited studies of pemphigoid diseases originated.

The distribution of the countries in the top 100 studies is presented in Figure 3. In total, 15 countries contributed to the top 100 studies. Generally, studies on high-citation pemphigoid diseases originated from Europe. Most of the studies were conducted in Germany (*n* = 22) and United States (*n* = 22), followed by France (*n* = 14), the UK (*n* = 10), and Japan (*n* = 10).

### Designs of studies and cited times

**FIGURE 4:**
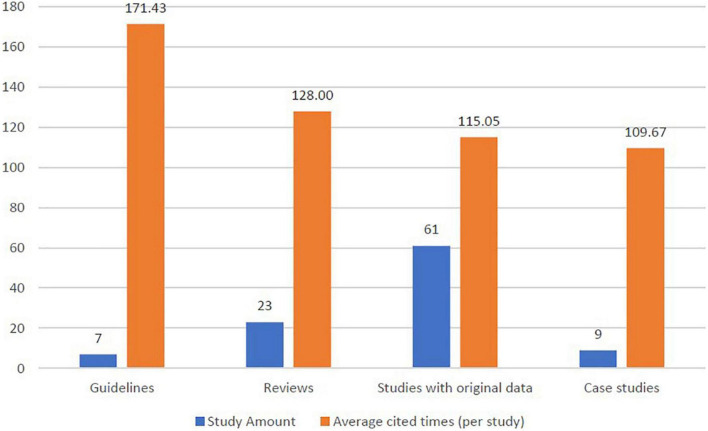
Distribution of study designs and cited amount (per study) in the top 100 publications of pemphigoid diseases.

More than 60% of the top 100 studies were studies with original data (no matter in research letter or in original article types). Furthermore, 30% of the studies were guidelines and narrative reviews (Figure 4). Study designs of studies with original data include randomized controlled trial (*n* = 3), prospective cohort studies (*n* = 5), retrospective cohort studies (*n* = 7), nested case-control studies (*n* = 1), prospective case-control studies (*n* = 1), retrospective case-control studies (*n* = 8), cross-sectional studies (*n* = 1) and clinical observations (*n* = 15).

Within the seven guidelines in the top 100 studies, the average cited times were numerically higher than other study designs. For guidelines, the average cited times was 171.43 times per study. For reviews, the average cited times was 128.00 times per study, while studies with original data presented the average cited times of 115.05 times per study.

### Research focus of studies

**FIGURE 5:**
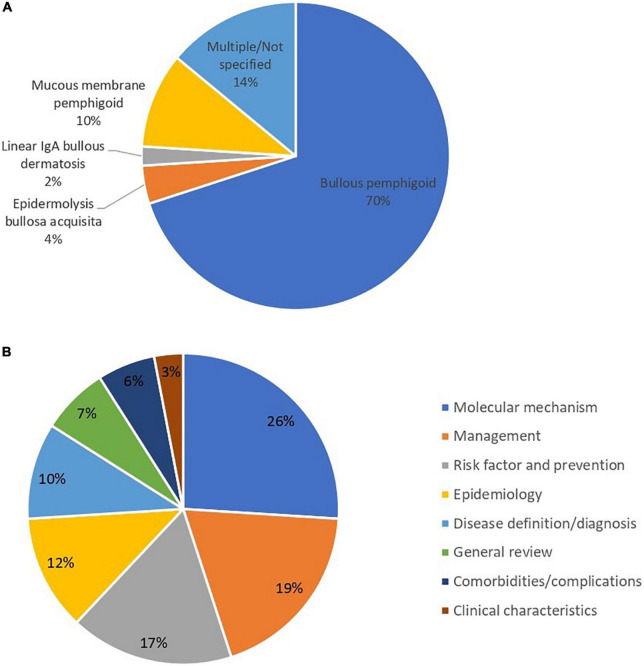
**(A)** Distribution of subcategories of pemphigoid diseases in the main focus of top 100 publications. **(B)** Main focus of the top 100 cited studies of pemphigoid diseases.

Figures 5A, B present the research focus of the top 100 studies. Among the top 100 studies, most of the studies focused on bullous pemphigoid (70%). For studies focusing on the mucous membrane pemphigoid (or cicatricial pemphigoid), the ratio was 10% (Figure 5A). In terms of fields of study, the most involved issues in the top 100 studies were regarding the molecular mechanism of pemphigoid diseases (10, 23, 26, 28, 29, 31, 41, 42, 45–47, 55, 59, 64, 70, 74, 75, 84, 87, 88, 92, 93, 95, 96, 99, 104) (26%), managements (7, 12, 34, 35, 37, 43, 51, 52, 54, 57, 65, 80, 82, 83, 85, 98, 102, 103, 105) (19%), and risk factors (11, 13, 14, 16, 19, 53, 60, 62, 66–68, 73, 77, 86, 89, 90, 97) (17%) (Figure 5B). Regarding the studies that focus on the field of risk factors, most of the studies focused on drug-induced pemphigoid diseases. Five of these studies (29%) described the association between DPP4 inhibitors and pemphigoid diseases (13, 14, 60, 89, 97) and another four studies (25%) described PD-1 or anti-PD-L1 inhibitor-induced pemphigoid diseases (19, 53, 68, 86). Among epidemiological studies, 7 studies (58%) described the incidence of bullous pemphigoid (17, 18, 38, 49, 61, 63, 69) and 6 studies (50%) described the mortality of bullous pemphigoid (18, 24, 38, 49, 63, 76).

## Discussion

The current study provided a bibliometric analysis of the 100 most cited studies in the field of pemphigoid diseases and revealed that issues regarding disease management, molecular mechanism of diseases, and risk factors of the disease were having the greatest impact in the field.

Within the top 100 studies, guidelines presented the highest amount per study. These guidelines included diagnosis, definition, and treatment of bullous pemphigoid and mucous membrane pemphigoid. A similar situation could also be observed for other types (Figure 5A). The fact that there are no highly cited guidelines and other research items for pemphigoid diseases other than bullous pemphigoid and mucous membrane pemphigoid should be carefully interpreted. The high number of bullous pemphigoid and mucous membrane pemphigoid could potentially be attributed to the fact that they were identified and researched earlier. However, many of the clinical guidelines regarding other pemphigoid diseases such as epidermolysis bullosa acquisita were relatively new and were published recently (106, 107).

According to the results of the current study, in the period between 2006 and 2011, studies of pemphigoid diseases with high citation were increasing. The observed trend could possibly be attributed to the improvement in the identification and treatment of pemphigoid diseases, especially in the field of molecular mechanisms. 7 out of 40 studies (17.5%) in the period were discussing the role of bullous pemphigoid antigens (including BP180 or BP230) in the detection of disease activity or diagnosis. Currently, BP180 and 230 are recognized as the two most important antigens in the pathogenesis of bullous pemphigoid. The two antigens could be related to IgG-related autoantibodies and consequently cause abnormal adhesion between the extracellular matrix and keratinocytes, contributing to the presence of blisters (108). Detection methods have also been widely discussed. The feasibility of enzyme-linked immunoassay analyzes (ELISA) in detecting BP180/230 in patients with bullous pemphigoid has been widely discussed in the past 20 years, and has been demonstrated by several high impact studies (21, 59).

Treatment for pemphigoid diseases was a growing issue in the field. According to the current study, nearly one fifth of the top-100 most cited studies focused on the topic of management of pemphigoid diseases. Before 2018, corticosteroids, doxycycline and biologic agents such as rituximab and omalizumab were reported to have benefit in management of pemphigoid diseases through different approaches (51, 65, 102, 105). Furthermore, in August 2018, dupilumab was time for the first reported to serve as a potential option for bullous pemphigoid management (109), and recent evidence had further demonstrated that dupilumab, rituximab, and omalizumab present similar treatment efficacy (110, 111). However, to date large scale real-world studies evaluating the long-term effect of patients with pemphigoid diseases using dupilumab is lacking and whether or not the prescription of biologic agents is associated with future immunological events is unknown. Moreover, efficacy and safety of some new potential options for treatment of pemphigoid diseases, such as the use of janus kinase inhibitor (JAKi), could also be evaluated in future studies. The involvement of JAK/STAT pathway and the potential effect of using JAKi for pemphigoid diseases has been recently reported by few studies (112, 113). However, the trend could not be observed in the top-100 most cited articles presented in the current study since related data was limited. In this case, knowledge gaps were warranted be filled, and future studies are suggested to focus on the long-term effect of using biologics and the effect of new options of medication.

Drug-induced pemphigoid diseases, especially bullous pemphigoid, was one of the most critical and most mentioned research focuses in the field. Our study revealed that more than 15% of the top 100 studies were discussing risk factors for pemphigoid diseases. Within them, 75% of the studies were evaluating the interaction between specific medication use and the influence of pemphigoid disease incidence or other associated adverse outcomes. Within the term 2001–2018, it was mentioned that dipeptidyl peptidase 4 inhibitors (DPP4i), anti-PD1 or PD-L1 and loop diuretics were mentioned to be associated with a higher risk of new-onset pemphigoid diseases (66, 68). Furthermore, according to a real-world study in Germany, the use of glucocorticoids was reported to increase mortality in patients with bullous pemphigoid (77). To provide an overview of the study trend in pemphigoid diseases in the last three years (2019-2021), we also list the top-5 most cited studies in 2019 (114–118), 2020 (109, 119–122) and 2021 (123–127) (Supplementary Table 1). Within these recent studies most cited, drug-induced pemphigoid diseases (especially bullous pemphigoid) were highly concerned, for more than one-third of the most cited studies stated the correlation between drug/vaccine use and the risk of pemphigoid diseases. In the field of drug-induced pemphigoid diseases, most studies focused on the incidence of bullous pemphigoid. However, only few high-impact studies discussed the incidence of other subcategories of pemphigoid diseases and the evidence might be insufficient. The observed trend indicated that the interaction between drug use and pemphigoid diseases was gradually seizing attention from the academic community. Based on this trend in the study, future studies with greater scale were warranted to identify new risk factors for pemphigoid diseases and to clarify detailed molecular mechanisms on the pathophysiology of drug-induced pemphigoid diseases.

Limitations of the current study must be stated, and the results reported in bibliometric studies should be dialectically interpreted. Although bibliometric studies were extensively applied in research fields to identify research focus and trends (128, 129), the amount of study being cited might not be able to accurately represent its influence. The amount could be influenced by external factors that were not directly related to the research itself, such as publishers, journals, and the fame of the research team. However, the trend provided in the current study was not to determine whether or not an article have enough impact on the academic community. Instead, the intention of analyzing the top 100 most cited studies was to provide the academic community with an overview of the current research reign of pemphigoid diseases and serving as a foundation for future research focuses.

As a conclusion, this comprehensive bibliographic study of pemphigoid diseases provided an overview of current research focuses in the field. Topics such as disease management, molecular mechanism of pathogenesis, and drug-inducing pemphigoid diseases were highly mentioned in previous studies. For researchers and clinicians, the researching trend and study focus in the top-100 cited studies which was presented in the tables of our current study could serve as a potential reference for future investigation and patient management.

## Data availability statement

The original contributions presented in this study are included in the article/Supplementary material, further inquiries can be directed to the corresponding authors.

## Author contributions

S-YG, S-CH, W-JW, T-MC, and H-CC: study conception and design. S-YG and S-CH: data acquisition. S-YG, S-CH, T-MC, and H-CC: data analysis and demonstration. All authors involved in original draft preparation, drafting or revising the manuscript, and approved the submitted version.
