# Supplementary material for: Researching trends in pemphigoid diseases: A bibliometric study of the top 100 most cited publications
Source: Front Med (Lausanne). 2023 Jan 9;9:1088083. doi: 10.3389/fmed.2022.1088083 (PMC9868262; doi:10.3389/fmed.2022.1088083)
Supplement: Supplementary file 1 [file Table_1.pdf]

Table S1. Detailed information of the top 5 most cited studies in 2019, 2020 and 2021 (in the sequence from most cited to least cited)

2019:

	Title	Year of publication	First author	Nation	Journal	IF 2021	citation amount	Study type	Detailed	Subgroup	Main focus	Open access/Subscription only	Number of Reference
1	Bullous pemphigoid.	2019	Miyamoto D	Brazil	Anais Brasileiros de Dermatologia	1.58	55	Review	Narrative	BP	General	Open access	(117)
2	Rituximab and Omalizumab for the Treatment of Bullous Pemphigoid: A Systematic Review of the Literature	2019	Kremer N	Israel	American Journal of Clinical Dermatology	7.4	53	Review	Systematic	BP	Treatment	Subscription only	(115)
3	Association of Dipeptidyl Peptidase 4 Inhibitor Use With Risk of	2019	Lee SG	Korea	JAMA Dermatology	11.82	53	Original	Retrospective case control	BP	Risk factor	Open access	(116)

	Bullous Pemphigoid in Patients With Diabetes												
4	Epidermolysis Bullosa Acquisita: The 2019 Update	2019	Koga H	Japan	Frontiers in Medicine	5.058	51	Review	Narrative	EBA	General	Open access	(114)
5	Higher Frequency of Dipeptidyl Peptidase-4 Inhibitor Intake in Bullous Pemphigoid Patients than in the French General Population	2019	Plaquevent M	French	Journal of Investigative Dermatology	7.59	49	Original	Retrospective case control	BP	Risk factor	Open access	(118)

2020

	Title	Year of publication	First author	Nation	Journal	IF 2021	citation amount	Study type	Detailed	Subgroup	Main focus	Open access/Subscription only	Number of Reference
1	Dupilumab as a novel therapy for bullous pemphigoid: A multicenter case series	2020	Abdat R	US	Journal of the American Academy of Dermatology	15.487	52	Case	Case series	BP	Treatment	Subscription only	(109)
2	Pathophysiologic mechanisms of itch in bullous pemphigoid.	2020	Hashimoto T	Japan	Journal of the American Academy of Dermatology	15.487	37	Review	Narrative	BP	Molecular mechanism	Subscription only	(120)
3	Autoimmune bullous skin diseases, pemphigus and pemphigoid	2020	Egami S	Japan	Journal of Allergy and Clinical Immunology	14.29	35	Review	Narrative	Multiple	General	Subscription only	(119)
4	Association Between Medication Use and Bullous Pemphigoid A Systematic Review and Meta-analysis	2020	Liu SD	Taiwan	JAMA Dermatology	11.82	29	Review	Meta analysis	BP	Risk factor	Open access	(121)

5	A Systematic Review of Drug-associated Bullous Pemphigoid	2020	Verheyden M	Australia	Acta Dermato-Venereologica	3.875	29	Review	Systematic review	BP	Risk factor	Open access	(122)

2021

	Title	Year of publication	First author	Nation	Journal	IF 2021	citation amount	Study type	Detailed	Subgroup	Main focus	Open access/Subscription only	Number of Reference
1	The first dose of COVID-19 vaccine may trigger pemphigus and bullous pemphigoid flares: is the second dose therefore contraindicated?	2021	Damiani G	Italy	Journal of the European Academy of Dermatology and Venereology	9.228	34	Case study	Case report	BP	Risk factor	Open access	(123)
2	Incidence, prevalence and mortality of bullous pemphigoid in England 1998-2017: a population-based cohort study	2021	Persson M	UK	British Journal of Dermatology	11.113	30	Original	Retrospective cohort	BP	Epidemiology	Subscription only	(126)
3	Dipeptidyl-peptidase IV inhibitor-associated bullous pemphigoid: A	2021	Kridin K	Israel	Journal of the American Academy of Dermatology	14.29	23	Review	Meta analysis	BP	Risk factor	Subscription only	(125)

	systematic review and meta-analysis												
4	European Guidelines (S3) on diagnosis and management of mucous membrane pemphigoid, initiated by the European Academy of Dermatology and Venereology - Part II	2021	Schmidt E	Germany	Journal of the European Academy of Dermatology and Venereology	9.228	20	Guideline	Guideline	MMP	Treatment	Open access	(127)
5	Updated international expert recommendations for the management of autoimmune bullous diseases during the COVID-19 pandemic	2021	Kasperkiewicz M	US	Journal of the European Academy of Dermatology and Venereology	9.228	18	Guideline	Guideline	Multiple	Treatment	Open access	(124)
