# Supplementary material for: Researching trends in pemphigoid diseases: A bibliometric study of the top 100 most cited publications
Source: Front Med (Lausanne). 2023 Jan 9;9:1088083. doi: 10.3389/fmed.2022.1088083 (PMC9868262; doi:10.3389/fmed.2022.1088083)
Supplement: Supplementary file 2 [file Table_2.pdf]

Table S2.

Detailed information of the top 100 most cited studies regarding pemphigoid diseases of all time (in the sequence from most cited to least cited)

	Title	Year of publication	First author	Country of origin	Journal	IF 2021	Cited times	Study type	Subgroup	Main focus	Open access/Subscription only	Number of Reference
1	Pemphigoid diseases	2013	Schmidt E	Germany	Lancet	202.731	581	R*	Multiple	general	Subscription only	(2)
2	The first international consensus on mucous membrane pemphigoid - Definition, diagnostic criteria, pathogenic factors, medical treatment, and prognostic indicators	2002	Chan LS	US	Archives of Dermatology**	11.816	476	G	MMP	definition/diagnosis	Subscription only	(20)
3	Bullous pemphigoid and pemphigus vulgaris - incidence and mortality in the UK: population based cohort study	2008	Langan SM	UK	British Medical Journal	93.333	402	O	BP	epidemiology/mortality	Open access	(63)
4	A comparison of oral and topical corticosteroids in	2002	Joly P	France	The New England Journal of Medicine	176.079	395	O	BP	management	Subscription only	(52)

	patients with bullous pemphigoid											
5	Incidence and Mortality of Bullous Pemphigoid in France	2012	Joly P	France	Journal of Investigative Dermatology	7.59	210	O	BP	epidemiology/mortality	Open access	(49)
6	Definitions and outcome measures for bullous pemphigoid: recommendations by an international panel of experts	2012	Murrell DF	Australia	Journal of the American Academy of Dermatology	15.487	190	G	BP	definition/diagnosis	Open access	(71)
7	Risk Factors for Bullous Pemphigoid in the Elderly: A Prospective Case-Control Study	2011	Bastuji-Garin S	France	Journal of Investigative Dermatology	7.59	187	O	BP	risk factor and prevention	Open access	(11)
8	Management of bullous pemphigoid: the European Dermatology Forum consensus in collaboration with the European Academy of	2015	C Feliciani	Italy	British Journal of Dermatology	11.113	186	G	BP	management	Subscription only	(35)

	Dermatology and Venereology											
9	Autoimmune Bullous Skin Disorders with Immune Checkpoint Inhibitors Targeting PD-1 and PD-L1	2016	Naidoo J	US	Cancer Immunology Research	12.02	180	C	BP	risk factor and prevention	Open access	(73)
10	Incidence of bullous pemphigoid and pemphigus in Switzerland: a 2-year prospective study	2009	Marazza G	Switzerland	British Journal of Dermatology	11.113	178	O	BP	epidemiology/mortality	Subscription only	(69)
11	Prospective analysis of the incidence of autoimmune bullous disorders in Lower Franconia, Germany	2009	Bertram F	Germany	Journal Der Deutschen Dermatologischen Gesellschaft	5.231	175	O	Multiple	epidemiology/mortality	Subscription only	(17)
12	BP180 ELISA using bacterial recombinant NC16a protein as a diagnostic and monitoring tool for bullous pemphigoid	2002	Kobayashi M	Japan	Journal of Dermatological Science	5.408	167	O	BP	molecular mechanism	Subscription only	(59)

13	A comparison of two regimens of topical corticosteroids in the treatment of patients with bullous pemphigoid: a multicenter randomized study	2009	Joly P	France	Journal of Investigative Dermatology	7.59	155	O	BP	management	Open access	(51)
14	Mechanisms of Disease: Pemphigus and Bullous Pemphigoid	2016	Hammers, CM	US	Annual Review of Pathology: Mechanisms of Disease	32.35	152	R	BP	molecular mechanism	Open access	(41)
15	Drug-induced pemphigoid: a review of the literature	2014	Stavropoulos PG	Greece	Journal of the European Academy of Dermatology and Venereology	9.228	151	R	BP	risk factor and prevention	Subscription only	(90)
16	Anti-epiligrin cicatricial pemphigoid and relative risk for cancer	2001	Egan CA	US	Lancet	202.731	150	C	MMP	comorbidities and complications	Subscription only	(32)
17	The Relationship between Neurological Disease and Bullous	2011	Langan SM	UK	Journal of Investigative Dermatology	7.59	146	O	BP	risk factor and prevention	Open access	(62)

	Pemphigoid: A Population-Based Case-Control Study											
18	Severity and phenotype of bullous pemphigoid relate to autoantibody profile against the NH2- and COOH-terminal regions of the BP180 ectodomain	2002	Hofmann S	Germany	Journal of Investigative Dermatology	7.59	145	O	BP	management	Open access	(43)
19	Rituximab in autoimmune bullous diseases: mixed responses and adverse effects	2007	Schmidt E	Germany	British Journal of Dermatology	11.113	138	O	Multiple	management	Open access	(83)
20	Risk factors for lethal outcome in patients with bullous pemphigoid - Low serum albumin level, high dosage of glucocorticosteroids, and old age	2002	Rzany B	Germany	Archives of Dermatology*	11.816	138	O	BP	risk factor and prevention	Open access	(77)

21	Autoantibody Profile Differentiates between Inflammatory and Noninflammatory Bullous Pemphigoid	2016	Izumi Kentaro	Japan	Journal of Investigative Dermatology	7.59	137	O	BP	molecular mechanism	Open access	(47)
22	Immunopathology and molecular diagnosis of autoimmune bullous diseases	2007	Mihai S	Germany	Journal of Cellular and Molecular Medicine	5.295	132	R	Multiple	molecular mechanism	Open access	(70)
23	The pathophysiology of bullous pemphigoid	2007	Kasperkiewicz M	Germany	Clinical Reviews in Allergy & Immunology	10.817	129	R	BP	molecular mechanism	Subscription only	(55)
24	Multicenter prospective study of the humoral autoimmune response in bullous pemphigoid	2008	Di Zenzo G	Italy	Clinical Immunology	10.19	128	O	BP	definition/diagnosis	Subscription only	(30)
25	T cell control in autoimmune bullous skin disorders	2006	Michael Hertl	Germany	Journal of Clinical Investigation	19.456	127	R	Multiple	molecular mechanism	Open access	(42)
26	Mucous membrane pemphigoid and pseudopemphigoid	2004	Thorne JE	US	Ophthalmology	14.277	124	O	MMP	comorbidities and complications	Subscription only	(94)

27	Modern diagnosis of autoimmune blistering skin diseases	2010	Schmidt E	Germany	Autoimmunity Reviews	17.39	122	R	Multiple	definition/diagnosis	Subscription only	(85)
28	Prediction of survival for patients with bullous pemphigoid - A prospective study	2005	Joly P	France	Archives of Dermatology*	11.816	120	O	BP	epidemiology/mortality	Open access	(50)
29	Comorbidity profiles among patients with bullous pemphigoid: a nationwide population-based study	2011	Chen YJ	Taiwan	British Journal of Dermatology	11.113	119	O	BP	comorbidities and complications	Subscription only	(22)
30	Bullous pemphigoid: Etiology, pathogenesis, and inducing factors: Facts and controversies	2013	Lo Schiavo	Italy	Clinics in Dermatology	2.797	115	R	BP	risk factor and prevention	Subscription only	(67)
31	Bullous Pemphigoid: A Review of its Diagnosis, Associations and Treatment	2017	Bernard P	France	American Journal of Clinical Dermatology	6.233	114	R	BP	general	Subscription only	(15)

32	Anti-laminin gamma-1 pemphigoid	2009	Dainichi T	Japan	Proceedings of the National Academy of Sciences of the United States of America	12.779	112	O	Multiple	molecular mechanism	Open access	(26)
33	Autoimmune bullous skin diseases. Part 1: Clinical manifestations	2011	Kneisel, A	Germany	Journal Der Deutschen Dermatologischen Gesellschaft	5.231	111	R	Multiple	clinical characteristics	Subscription only	(58)
34	Enzyme-linked immunosorbent assay using multimers of the 16th non-collagenous domain of the BP180 antigen for sensitive and specific detection of pemphigoid autoantibodies	2007	Sitaru C	Germany	Experimental Dermatology	4.511	111	O	Multiple	molecular mechanism	Subscription only	(87)
35	Bullous pemphigoid and dipeptidyl peptidase IV inhibitors: a case-noncase study in the	2016	Bene J	France	British Journal of Dermatology	11.113	109	O	BP	risk factor and prevention	Subscription only	(13)

	French Pharmacovigilance Database											
36	Demonstration of Epitope-Spreading Phenomena in Bullous Pemphigoid: Results of a Prospective Multicenter Study	2011	Di Zenzo G	Italy	Journal of Investigative Dermatology	7.59	109	O	BP	molecular mechanism	Open access	(29)
37	Annual incidence and mortality of bullous pemphigoid in the Grampian Region of North-east Scotland	2005	Gudi VS	UK	British Journal of Dermatology	11.113	108	O	BP	epidemiology/mortality	Subscription only	(38)
38	Interventions for bullous pemphigoid	2010	Kirtschig G	Netherlands	Cochrane Database of Systematic Reviews	12.008	107	R	BP	management	Open access	(57)
39	Enzyme-linked immunosorbent assay using bacterial recombinant proteins of human BP230 as a	2006	Yoshida M	Japan	Journal of Dermatological Science	5.408	107	O	BP	molecular mechanism	Subscription only	(104)

	diagnostic tool for bullous pemphigoid											
40	Doxycycline versus prednisolone as an initial treatment strategy for bullous pemphigoid: a pragmatic, non-inferiority, randomised controlled trial	2017	Williams, HC	UK	Lancet	202.731	106	O	BP	management	Open access	(102)
41	Epidermolysis bullosa acquisita	2012	Gupta R	US	Clinics in Dermatology	2.797	106	R	EBA	general	Open access	(40)
42	Identification of a potential effector function for IgE autoantibodies in the organ-specific autoimmune disease bullous pemphigoid	2003	Dimson OG	US	Journal of Investigative Dermatology	7.59	106	O	BP	molecular mechanism	Open access	(31)
43	Autoantibodies to type VII collagen mediate Fc gamma-dependent neutrophil activation and induce dermal-	2002	Sitaru C	Germany	The American Journal of Pathology	5.77	106	O	EBA	molecular mechanism	Open access	(88)

	epidermal separation in cryosections of human skin										
44	Combination of Rituximab and Intravenous Immunoglobulin for Recalcitrant Ocular Cicatricial Pemphigoid A Preliminary Report	2010	Foster CS	US	Ophthalmology	14.277	104	O	MMP	management	Subscription only	(37)
45	Pathogenicity of IgE in autoimmunity: Successful treatment of bullous pemphigoid with omalizumab	2009	Fairley JA	US	Journal of Allergy and Clinical Immunology	14.29	103	C	BP	management	Open access	(34)
46	Immunosuppressive therapy for ocular mucous membrane pemphigoid strategies and outcomes	2008	Saw VPJ	UK	Ophthalmology	14.277	102	C	MMP	management	Subscription only	(80)
47	U-serrated immunodeposition pattern differentiates type VII collagen	2004	Vodegel RM	Netherlands	British Journal of Dermatology	11.113	102	O	Multiple	definition/diagnosis	Open access	(100)

	targeting bullous diseases from other subepidermal bullous autoimmune diseases											
48	The Association of Bullous Pemphigoid With Cerebrovascular Disease and Dementia A Case-Control Study	2010	Taghipour K	UK	Archives of Dermatology*	11.816	101	O	BP	comorbidities and complications	Subscription only	(91)
49	Drug-induced bullous pemphigoid in diabetes mellitus patients receiving dipeptidyl peptidase-IV inhibitors plus metformin	2012	Skandalis K	Greece	Journal of the European Academy of Dermatology and Venereology	9.228	100	C	BP	risk factor and prevention	Subscription only	(89)
50	Bullous pemphigoid in infancy: Clinical and epidemiologic characteristics	2008	Waisbourd-Zinman O	Israel	Journal of the American Academy of Dermatology	15.487	100	R	BP	clinical characteristics	Subscription only	(101)
51	Dipeptidyl peptidase IV inhibitors, a risk factor for bullous pemphigoid:	2018	Benzaquen M	France	Journal of the American Academy of Dermatology	15.487	98	O	BP	risk factor and prevention	Open access	(14)

	Retrospective multicenter case-control study from France and Switzerland											
52	Type VII Collagen: The Anchoring Fibril Protein at Fault in Dystrophic Epidermolysis Bullosa	2010	Chung HJ	US	Dermatologic Clinics	3.645	98	R	EBA	molecular mechanism	Open access	(23)
53	Neurological disorders in patients with bullous pemphigoid	2007	Cordel N	France	Dermatology	5.197	98	O	BP	comorbidities and complications	Subscription only	(25)
54	Prevalence and Age Distribution of Pemphigus and Pemphigoid Diseases in Germany	2016	Hubner F	Germany	Journal of Investigative Dermatology	7.59	97	L	Multiple	epidemiology/mortality	Open access	(44)
55	Omalizumab therapy for bullous pemphigoid	2014	Yu KK	US	Journal of the American Academy of Dermatology	15.487	97	C	BP	management	Open access	(105)

56	BP230- and BP180-specific auto-antibodies in bullous pemphigoid	2004	Thoma-Uszynski S	Germany	Journal of Investigative Dermatology	7.59	97	O	BP	molecular mechanism	Open access	(92)
57	Bullous disorders associated with anti-PD-1 and anti-PD-L1 therapy: A retrospective analysis evaluating the clinical and histopathologic features, frequency, and impact on cancer therapy	2018	Siegel, J	US	Journal of the American Academy of Dermatology	15.487	95	C	Multiple	risk factor and prevention	Subscription only	(86)
58	Diagnosis and classification of pemphigus and bullous pemphigoid	2014	Kershenovich R	Israel	Autoimmunity Reviews	17.39	95	R	BP	definition/diagnosis	Subscription only	(56)
59	Linear immunoglobulin A bullous dermatosis	2012	Fortuna G	US	Clinics in Dermatology	2.797	95	R	IgAB	general	Subscription only	(36)
60	A comparison of oral methylprednisolone plus azathioprine or	2007	Beissert S	Germany	Archives of Dermatology*	11.816	94	O	BP	management	Open access	(12)

	mycophenolate mofetil for the treatment of bullous pemphigoid											
61	IgG from Patients with Bullous Pemphigoid Depletes Cultured Keratinocytes of the 180-kDa Bullous Pemphigoid Antigen (Type XVII Collagen) and Weakens Cell Attachment	2009	Iwata H	Japan	Journal of Investigative Dermatology	7.59	93	O	BP	molecular mechanism	Open access	(45)
62	Correlation of IgE autoantibody to BP180 with a severe form of bullous pemphigoid	2008	Iwata Y	Japan	Archives of Dermatology*	11.816	93	O	BP	molecular mechanism	Open access	(46)
63	Association of Bullous Pemphigoid With Dipeptidyl-Peptidase 4 Inhibitors in Patients With Diabetes: Estimating the Risk of the New Agents and	2018	Kridin K	Israel	JAMA Dermatology	11.816	93	O	BP	risk factor and prevention	Open access	(60)

	Characterizing the Patients											
64	Bullous pemphigoid: From the clinic to the bench	2012	Di Zenzo	Italy	Clinics in Dermatology	2.797	92	R	BP	general	Subscription only	(27)
65	Cicatricial pemphigoid: IgA and IgG autoantibodies target epitopes on both intra- and extracellular domains of bullous pemphigoid antigen 180	2001	Schmidt E	Germany	British Journal of Dermatology	11.113	92	O	MMP	molecular mechanism	Subscription only	(84)
66	A review of bullous pemphigoid associated with PD-1 and PD-L1 inhibitors	2018	Lopez AT	US	International Journal of Dermatology	3.204	91	R	BP	risk factor and prevention	Subscription only	(68)
67	British Association of Dermatologists' guidelines for the management of bullous pemphigoid 2012	2012	Venning VA	UK	British Journal of Dermatology	11.113	90	G	BP	management	Open access	(98)

68	Rituximab for treatment-refractory pemphigus and pemphigoid: A case series of 17 patients	2011	Kasperkiewicz M	Germany	Journal of the American Academy of Dermatology	15.487	90	C	BP	management	Subscription only	(54)
69	Autoimmune dermatologic toxicities from immune checkpoint blockade with anti-PD-1 antibody therapy: a report on bullous skin eruptions	2016	Jour G	US	Journal of Cutaneous Pathology	1.458	89	C	BP	risk factor and prevention	Subscription only	(53)
70	Definitions and outcome measures for mucous membrane pemphigoid: Recommendations of an international panel of experts	2015	Murrell DF	Australia	Journal of the American Academy of Dermatology	15.487	89	G	MMP	definition/diagnosis	Open access	(72)
71	The Growing Incidence of Bullous Pemphigoid: Overview	2018	Kridin K	Israel	Frontiers in Medicine	5.058	88	R	BP	epidemiology/mortality	Open access	(61)

	and Potential Explanations											
72	Autoimmune Subepidermal Bullous Diseases of the Skin and Mucosae: Clinical Features, Diagnosis, and Management	2018	Amber KT	US	Clinical Reviews in Allergy & Immunology	10.817	88	R	Multiple	general	Subscription only	(9)
73	Geographic variations in epidemiology of two autoimmune bullous diseases: pemphigus and bullous pemphigoid	2015	Alpsoy E	Turkey	Archives of Dermatological Research	3.033	88	R	BP	epidemiology/mortality	Subscription only	(8)
74	A case of bullous pemphigoid in a patient with metastatic melanoma treated with pembrolizumab	2015	Carlos G	Australia	Melanoma Research	3.199	87	C	BP	risk factor and prevention	Subscription only	(19)
75	Risk Factors for Relapse in Patients With Bullous Pemphigoid in Clinical Remission A	2009	Bernard P	France	Archives of Dermatology*	11.816	86	O	BP	risk factor and prevention	Subscription only	(16)

	Multicenter, Prospective, Cohort Study											
76	Rituximab in refractory autoimmune bullous diseases	2006	Schmidt E	Germany	Clinical and Experimental Dermatology	4.481	86	C	Multiple	management	Subscription only	(82)
77	Correlation of clinical severity and ELISA indices for the NC16A domain of BP180 measured using BP180 ELISA kit in bullous pemphigoid	2005	Tsuji-Abe Y	Japan	Journal of Dermatological Science	5.408	86	O	BP	molecular mechanism	Subscription only	(95)
78	S2k guideline for the diagnosis of pemphigus vulgaris/foliaceus and bullous pemphigoid	2015	Schmidt E	Germany	Journal Der Deutschen Dermatologischen Gesellschaft	5.231	85	G	BP	definition/diagnosis	Subscription only	(81)
79	Linear IgA bullous dermatosis	2001	Guide SV	US	Clinics in Dermatology	2.797	85	R	IgAB	general	Subscription only	(39)
80	Bullous pemphigoid antigen II (BP180) and its soluble extracellular domains	2006	Oyama N	UK	British Journal of Dermatology	11.113	84	O	MMP	molecular mechanism	Subscription only	(75)

	are major autoantigens in mucous membrane pemphigoid: the pathogenic relevance to HLA class II alleles and disease severity											
81	Guidelines for the management of bullous pemphigoid	2002	Wojnarowska F	UK	British Journal of Dermatology	11.113	84	G	BP	management	Subscription only	(103)
82	Autoreactive T and B cells from bullous pemphigoid (BP) patients recognize epitopes clustered in distinct regions of BP180 and BP230	2006	Thoma-Uszynski S	Germany	Journal of Immunology	5.426	81	O	BP	molecular mechanism	Open access	(93)
83	Correlation of Serum Levels of IgE Autoantibodies Against BP180 With Bullous Pemphigoid Disease Activity	2017	van Beek N	Germany	JAMA Dermatology	11.816	80	O	BP	molecular mechanism	Open access	(96)

84	Respective contribution of neutrophil elastase and matrix metalloproteinase 9 in the degradation of BP180 (type XVII collagen) in human bullous pemphigoid	2001	Verraes S	France	Journal of Investigative Dermatology	7.59	80	O	BP	molecular mechanism	Open access	(99)
85	Vildagliptin Significantly Increases the Risk of Bullous Pemphigoid: A Finnish Nationwide Registry Study	2018	Varpuluoma O	Finland	Journal of Investigative Dermatology	7.59	78	L	BP	risk factor and prevention	Open access	(97)
86	Rituximab for Patients With Refractory Mucous Membrane Pemphigoid	2011	Le Roux-Villet C	France	Archives of Dermatology*	11.816	78	O	MMP	management	Subscription only	(65)
87	Intravenous immunoglobulin therapy for patients with bullous pemphigoid	2001	Ahmed AR	US	Journal of the American Academy of Dermatology	15.487	78	O	BP	management	Subscription only	(7)

	unresponsive to conventional immunosuppressive treatment											
88	Update on the pathogenesis of bullous pemphigoid: An autoantibody-mediated blistering disease targeting collagen XVII	2014	Nishie W	Japan	Journal of Dermatological Science	5.408	77	R	BP	molecular mechanism	Subscription only	(74)
89	Mortality of bullous pemphigoid: an evaluation of 223 patients and comparison with the mortality in the general population in the United States	2008	Parker SRS	US	Journal of the American Academy of Dermatology	15.487	77	O	BP	epidemiology/mortality	Subscription only	(76)
90	Innate Immune Cell-Produced IL-17 Sustains Inflammation in Bullous Pemphigoid	2014	Le Jan S	France	Journal of Investigative Dermatology	7.59	76	O	BP	molecular mechanism	Open access	(64)

91	The Associations Between Bullous Pemphigoid and Drug Use A UK Case-Control Study	2013	Lloyd-Lavery A	UK	JAMA Dermatology	11.816	76	O	BP	risk factor and prevention	Open access	(66)
92	Bullous pemphigoid and internal diseases - A case-control study	2010	Jedlickova H	Czech	European Journal of Dermatology	2.805	76	O	BP	comorbidities and complications	Subscription only	(48)
93	Anti-epiligrin cicatricial pemphigoid - Clinical findings, immunopothogenesis, and significant associations	2003	Egan CA	US	Medicine	1.817	76	O	MMP	clinical characteristics	Subscription only	(33)
94	Lesional Th17 cells and regulatory T cells in bullous pemphigoid	2011	Arakawa M	Japan	Experimental Dermatology	4.511	75	L	BP	molecular mechanism	Subscription only	(10)
95	Characterization of the anti-BP180 autoantibody reactivity profile and epitope mapping in bullous pemphigoid patients	2004	Di Zenzo	Italy	Journal of Investigative Dermatology	7.59	75	O	BP	molecular mechanism	Open access	(28)

96	Incidence of bullous pemphigoid and mortality of patients with bullous pemphigoid in Olmsted County, Minnesota, 1960 through 2009	2014	Brick KE	US	Journal of the American Academy of Dermatology	15.487	73	O	BP	epidemiology/mortality	Open access	(18)
97	Comparative study of direct and indirect immunofluorescence and of bullous pemphigoid 180 and 230 enzyme-linked immunosorbent assays for diagnosis of bullous pemphigoid	2013	Sardy M	Germany	Journal of the American Academy of Dermatology	15.487	73	O	BP	definition/diagnosis	Subscription only	(79)
98	Development of NC1 and NC2 domains of Type VII collagen ELISA for the diagnosis and analysis of the time course of	2011	Saleh MA	Japan	Journal of Dermatological Science	5.408	73	O	EBA	definition/diagnosis	Subscription only	(78)

	epidermolysis bullosa acquisita patients											
99	Usefulness of BP230 and BP180-NC16a enzyme-linked immunosorbent assays in the initial diagnosis of bullous pemphigoid: a retrospective study of 138 patients	2011	Charneux J	France	Archives of Dermatology*	11.816	73	O	BP	definition/diagnosis	Open access	(21)
100	Mortality rate of bullous pemphigoid in a US medical center	2004	Colbert, RL	US	Journal of Investigative Dermatology	7.59	73	O	BP	epidemiology/mortality	Open access	(24)

** R, reviews; O, original articles; G, guidelines; C, case studies; L, letters with original data; BP, bullous pemphigoid; MMP, mucous membrane pemphigoid; EBA, epidermolysis bullosa acquisita; IgAb, Linear IgA bullous dermatoses*

*** The journal "Archives of Dermatology" had changed its name into "JAMA Dermatology" in 2013*
